# Antitumor activity of dual blockade of PD-L1 and MEK in NSCLC patients derived three-dimensional spheroid cultures

**DOI:** 10.1186/s13046-019-1257-1

**Published:** 2019-06-13

**Authors:** Carminia Maria Della Corte, Giusi Barra, Vincenza Ciaramella, Raimondo Di Liello, Giovanni Vicidomini, Silvia Zappavigna, Amalia Luce, Marianna Abate, Alfonso Fiorelli, Michele Caraglia, Mario Santini, Erika Martinelli, Teresa Troiani, Fortunato Ciardiello, Floriana Morgillo

**Affiliations:** 1 Oncologia Medica, Dipartimento di Medicina di Precisione, Università degli studi della Campania “Luigi Vanvitelli”, Via S. Pansini 5, 80131 Naples, Italy; 2 Chirurgia Toracica, Dipartimento di Scienze Mediche Traslazionali, Università degli studi della Campania “Luigi Vanvitelli”, Napoli, Italy; 3 Biochimica Generale, Dipartimento di Medicina di Precisione, Università degli studi della Campania “Luigi Vanvitelli”, Napoli, Italy

**Keywords:** MEK, PD-L1, Lung cancer, Organoid cultures

## Abstract

**Background:**

Anti-PD-1/PD-L1 drugs are effective as monotherapy in a proportion of NSCLC patients and there is a strong rationale for combining them with targeted therapy. Inhibition of MAPK pathway may have pleiotropic effects on the microenvironment. This work investigates the efficacy of combining MEK and PD-L1 inhibition in pre-clinical and ex-vivo NSCLC models.

**Methods:**

We studied the effects of MEK inhibitors (MEK-I) on PD-L1 and MCH-I protein expression and cytokine production in vitro in NSCLC cell lines and in PBMCs from healthy donors and NSCLC patients, the efficacy of combining MEK-I with anti-PD-L1 antibody in ex-vivo human spheroid cultures obtained from fresh biopsies from NSCLC patients in terms of cell growth arrest, cytokine production and T-cell activation by flow cytometry.

**Results:**

MEK-I modulates *in–vitro* the immune micro-environment through a transcriptionally decrease of PD-L1 expression, enhance of MHC-I expression on tumor cells, increase of the production of several cytokines, like IFNγ, IL-6, IL-1β and TNFα. These effects trigger a more permissive anti-tumor immune reaction, recruiting immune cells to the tumor sites. We confirmed these data on ex-vivo human spheroids, showing a synergism of MEK and PD-L1 inhibition as result of both direct cancer cell toxicity of MEK-I and its immune-stimulatory effect on cytokine secretion profile of cancer cells and PBMCs with the induction of the ones that sustain an immune-reactive and inflammatory micro-environment.

**Conclusions:**

Our work shows the biological rationale for combining immunotherapy with MEK-I in a reproducible ex-vivo 3D-culture model, useful to predict sensitivity of patients to such therapies.

**Electronic supplementary material:**

The online version of this article (10.1186/s13046-019-1257-1) contains supplementary material, which is available to authorized users.

## Background

Non-small cell lung cancer (NSCLC) accounts for ~ 85% of all lung cancers and is commonly diagnosed in advanced stage; even those patients undergoing potentially curative surgery can experience systemic relapse, within few years, suggesting the systemic nature of the disease [1]. During the last two decades progress has been made in developing targeted agents for the small subgroup of oncogene-addicted NSCLC, including EGFR mutated and ALK translocated patients, while cytotoxic chemotherapy has represented the only available treatment for all unselected NSCLC patients [2] until the introduction of immunotherapy. Only in recent years, additional understanding of the interaction between the immune system and tumor cells has led to the development of this new class of drugs with the goal to boost the host’s own immune response against cancer. Immunotherapies include immune checkpoint inhibitors, such as monoclonal antibodies directed against cytotoxic T-lymphocyte-associated antigen-4 (CTLA-4) and programmed cell death protein-1 (PD-1)/programmed cell death ligand-1 (PD-L1) pathway [3]. Anti-PD-1/PD-L1 agents have demonstrated in multiple phase I–III clinical trials a great efficacy in terms of significant durable tumor responses and survival benefit, with manageable toxicities, across different malignancies, including those ones traditionally defined as non-immunogenic, such as NSCLC [4, 5], and are currently approved worldwide as standard treatment for advanced NSCLC.

Among anti-PD-L1 drugs, atezolizumab (MPDL3280A) is an engineered IgG antibody, with a modified Fc domain that prevents antibody-dependent cell-mediated cytotoxicity, actually approved by FDA for second line treatment of NSCLC. This approval came from results of the randomized, open-label, phase III trial (OAK) [6]: atezolizumab, compared to the standard of care represented by docetaxel chemotherapy, prolonged the overall survival (OS), both in the intention to treat (ITT) population (median OS: 13.8 vs 9.6 months; hazard ratio [HR] 0.73, *p* = 0.0003) and in the PD-L1-positive (1/2/3 or IC1/2/3) population (median OS: 15.7 vs 10.3 months; HR 0.74; *p* = 0.0102), thus demonstrating a clinically relevant efficacy, regardless of PD-L1 expression.

On the other side, the MAPK signaling cascade is a key intracellular pathway that transduces physiologically multiple proliferative and differentiating signals from the extracellular environment [7, 8] and is often upregulated in tumorigenesis, leading to uncontrolled proliferation, invasion, metastasis and angiogenesis. Therapeutic inhibition of the MAPK pathway can be obtained with suppression of the key hubs MEK1 and MEK2 [7, 8]. Selumetinib is a potent and highly selective reversible MEK inhibitor (MEK-I), currently approved in combination with vemurafenib, a BRAF-inhibitor (BRAF-I), for advanced BRAF-mutated melanoma patients.

Preclinical models suggest that targeting MAPK pathway affects tumor growth in a broader way, being MAPK also implicated in immune resistance: MEK inhibition may represent a potential mechanism to convert otherwise resistant cancers by recruiting immune cells [9, 10] to the tumor sites. In melanoma patients, treatment with the combination of BRAF-I and MEK-I increased intra-tumor cytotoxic lymphocytes, as observed by Kakavand et al. in post-treatment biopsies [11].

Additionally, MEK-I seems to modulate the immune micro-environment enabling a more permissive immune reaction against the tumor, through different mechanisms: i) inhibition of vascular maturity and integrity and consequent higher immune infiltration in the tumor, ii) direct activation of neutrophils, antigen-presentation cells (APC) such as macrophage and dendritic cells, and of both T-cell subsets, CD8-positive cytotoxic and CD4-positive helper T-cells. All these effects may facilitate a better tumor recognition and killing by the immune system, particularly when those immune cells are activated by the concomitant treatment with an anti-PD-1/PD-L1 agent [12, 13].

In light of this rationale of synergism between these two classes of inhibitors, we plan to study the anti-tumor activity and the immune effects of the combination of atezolizumab, anti-PD-L1, and selumetinib, MEK-I, in preclinical and clinical derived models of NSCLC.

## Methods

All human samples and biopsies were collected after obtaining a written informed consensus from any patient and healthy donor, in accordance with the Declaration of Helsinki. The use of these samples for research purposes was approved by our local Ethical Committee. All below described methods were performed in accordance with guidelines and regulations.

### Cell lines and drugs

The human NSCLC cell lines were provided by American Type Culture Collection (ATCC, Manassas, VA, USA) and maintained in RPMI-1640 (Sigma-Aldrich) medium supplemented with 10% fetal bovine serum (FBS; Life Technologies, Gaithersburg, MD) in a humidified atmosphere with 5% CO2. The identity of all cell lines was confirmed by STR profiling (Promega) on an ad hoc basis prior to perform experiments.

Selumetinib (MEK-I, AZD6244) and atezolizumab were purchased from Selleck Chemicals, Munich, Germany. Avelumab, was provided by EMD Serono as a part of a Cooperative Research and Development agreement with our institution.

Primary antibodies for western blot analysis against phospho-MEK, MEK, phospho-MAPK44/42, MAPK44/42, PD-L1, phospho-STAT3 and MHC-I were obtained from Cell Signaling Technology; the following secondary antibodies from Bio-Rad were used: goat anti-rabbit IgG, rabbit anti-mouse IgG and monoclonal anti-β actin antibody from Sigma Chemical Co.

### Peripheral blood mononuclear cells (PBMCs) isolation and stimulation

PBMCs from healthy donors or NSCLC patients were isolated by Ficoll-Paque Plus (GE Healthcare). Isolated cells were grown for 24 h or 5 days, in complete medium composed by RPMI 1640 containing human AB serum (10%), Ultraglutamine I (1%), penicillin and streptomycin (1%) along with beads coated with anti-CD3 and anti-CD28 (Life Technologies) at a ratio of 1 bead per 10 cells. Cells were cultured in presence or absence of MEK-I selumetinib at 0.01 uM concentration.

### Quantitative real time PCR

Total RNA was extracted using Trizol reagent (Life Technologies). Reverse transcriptase reaction was carried out to convert 1 μg of isolated RNA into cDNA using sensi fast reverse transcriptase (bioline) according to the manufacturer instruction. Expression levels of genes encoding for: PD-L1, IFN-γ, IL-12, IL-1b, TNFα, IL-6, IL-10, TIM-3, CTLA-4, LAG-3 were analyzed using Real time quantitative PCR (RT-qPCR). Gene-specific primers were designed by using PRIMER EXPRESS software (Applied Biosystems). The primers used were: TIM3 FW: TACTGCCGGATCCAAAT; RV:TGACCTTGGCTGGTTTGATG; CTLA-4 FW: AAGGTGGAGCTCATGTACCC; RV: TCTGGGTTCCGTTGCCTATG; LAG3 FW: TGGGCACTACACCTGCCATA; RV: AGGATTTGGGAGTCACTGTGATG; IL-1B FW: GCTGATGGCCCTAAACAGATG; RV: TTGCTGTAGTGGTGGTCGGA; PD-L1 FW:CTGCACTTTTAGGAGATTAGATCCTG; RV:TGGGATGACCAATTCAGCTGTA; IFNG FW: ATGGCTGAACTGTCGCAAG; RV:TGCAGGCAGGACAACCATT; IL-12 FW:TTTATGATGGCCCTGTGCCT; RV: GGTCTTGAACTCCACCTGGTA; IL-0 FW:GGGAGAACCTGAAGACCCTC; RV:AAGAAATCGATGACAGCGCC; TNFα FW: AGCCCATGTTGTAGCAAACC; RV:CCAAAGTAGACCTGCCCAGA. Amplifications were done using the SYBR Green PCR Master Mix (Applied Biosystems). The thermal cycling conditions were composed of 50 °C for 2 min (stage 1) followed by a denaturation step at 95 °C for 10 min (stage 2) and then 40 cycles at 95 °C for 15 s and 60 °C for 1 min (stage 3). All samples were run in duplicate, in 25 μL reactions using a quant studio 7 flex (Applied Biosystems) and relative expression of genes was determined by normalizing to 18S, used as internal control gene; to calculate relative gene expression in value it was used the 2- ΔCt or 2- ΔΔCt method. Nonspecific signals caused by primer dimers were excluded by dissociation curve analysis and use of non-template controls.

### Western blot analysis

Protein lysates were obtained by homogenization in RIPA lyses buffer [0.1% sodium dodecylsulfate (SDS), 0,5% deoxycholate, 1% Nonidet, 100 mmol/L NaCl, 10 mmol/L Tris–HCl (pH 7.4), 0.5 mmol/L dithiotritol, and 0.5% phenylmethyl sulfonyl fluoride, protease inhibitor cocktail (Hoffmann-La Roche)] and clarification by centrifugation at 14,000 rpm for 15 min a 4 °C. Protein samples containing comparable amounts of proteins, estimated by a modified Bradford assay (Bio-Rad), were subjected to western blot and immune-complexes were detected with the enhanced chemiluminescence kit ECL plus, by Thermo Fisher Scientific (Rockford, IL) using the ChemiDoc (Bio-Rad). Each experiment was done in triplicate.

### Chip assay

Chromatin immunoprecipitation (ChIP) assay was performed as previously described with slight modification [14]. The major steps in the ChIP assay include the crosslinking of target protein to the chromatin DNA with formaldehyde, the breaking of the chromatin DNA into fragments (400–1200 bp), the immunoprecipitation (IP) of the protein-DNA complex with an antibody that recognizes the target protein. The DNA in IP product was amplified in PCR with the ChIP assay primers that are specific to the NF-κB binding site at − 316/− 15. The sequences of the primers specific to the promoter of PD-L1 gene are 5′- TGGACTGACATGTTTCACTTTCT − 3′(forward), and 5′-CAAGGCAGCAAATCCAGTTT-3′ (reverse). PCR products were analyzed on 2% agarose gel and images were analyzed with Storm 860 Molecular Imager scanner for densitometric measurements.

### Silencing

The small inhibitor duplex RNAs (siRNA) (ONtargetplus SMARTpool) siStat3 and siCONTROL NontargetingPool (no. D-001206-13-05), used as a negative (scrambled) control, were provided from Dharmacon (Lafayette, CO). Cells were transfected with 100 nM siRNAs using Dharmafect reagent following manufacturer’s instructions. The day before transfection, the cells were plated in 35 mm dishes at 40% of confluence in medium supplemented with 5% FBS without antibiotics. Where necessary, cells were treated with different compounds, as previously described; 24 and 48 h before harvesting and Western blot analysis were then performed.

### Flow cytometry

For FACS surface staining, cells were washed in staining buffer (SB) (2% FBS; 0,1% sodium azide in PBS) and after a blocking of 10 min with SB + Ab serum 20%, were stained for 30 min with mouse monoclonal antibodies. The antibodies used were: anti CD3, CD4, CD8, CD14, CD45, CD11C, EPCAM, PD-1, PD-L1, MHC-I, CD-107A (Miltenyi Biotec). Stained cells were washed 2 times, resuspended in SB and then acquired on a FACS ACCURI C6 (BD Biosciences). Analysis was conduced using accuri c6 software (BD Biosciences). The analysis of intracellular cytokine production was done after 6 h of stimulation with with phorbol 12-myristate 13-acetate (PMA, 10 ng/mL), Ionomycin (500 ng/mL) and Brefeldin A (BFA 10 μg/mL) (Sigma Aldrich) and the intracellular staining was performed incubating T cells with mouse monoclonal antibody IFNg (Miltenyi Biotech).

### Generation of ex vivo 3D cultures from patient samples

We developed a protocol for ex-vivo 3D cultures from lung cancer patient samples [15]. The protocol has been approved by our local Ethics Committee and all patients gave their written informed consent to the use of the tumor sample. All fresh tumor tissue samples were kept on ice and processed in sterile conditions on the day of collection. Tissue fragments were digested as previously described [16] in a 37 °C shaker at low to moderate speed (e.g. 200 rpm) for incubation time between 12 and 18 h and cells were separated with serial centrifugation. For 3D cultures, cells were seeded in matrigel in order to preserve three-dimensional structure.

### Cell viability assay

Cell viability was measured with the 3-(4,5-dimethylthiazol-2-yl)-2,5-diphenyltetrazolium bromide (MTT) assay the MTT assay, as previously described [17]. For 3D cultures, cells were extracted from matrigel with cold PBS-EDTA solution after the coloration with MTT and then lysed according to protocol instructions. IC_50_ were determined by interpolation from dose-response curves. Results represent the median of three separate experiments, each performed in quadruplicate. Synergism was calculated with the ComboSyn software, ComboSyn Inc., Paramus, NK. 07652 USA.

### Immunofluorescence

Organoids in matrigel were fixed for 20 min with a 4% paraformaldehyde (PFA) solution and made permeable for 10 min with 0.1% Triton X-100 in phosphate-buffered saline (PBS) at room temperature. Then organoids were incubated with a specific mouse monoclonal Ab raised against CD45 and cytokeratin (1:1000 in blocking solution, 3% BSA in TBS-Tween 0.1%, Sigma) for 2 h at 37 °C followed by revelation using Alexa Fluor 633-conjugated anti-rabbit immunoglobulin (Ig)G antibodies and Alexa Fluor 488-conjugated anti-rabbit IgG antibodies, respectively (Jackson Immunoresearch Laboratories, West Grove, PA, USA) at a dilution of 1:1000 for 1 h. The fluorescence was analyzed by an LSM-410 Zeiss confocal microscope.

### Statistical analysis

Statistical analysis was performed using Graphpad Prism software version 6.0 (Graphpad Software Inc., San Diego, CA, USA). Data were compared with One-way ANOVA statistical test followed by Tukey’s test. *P* values less than 0.05 were considered statistically significant.

## Results

### Role of MEK signal on PD-L1 expression on cancer cells

**Fig. 1:**
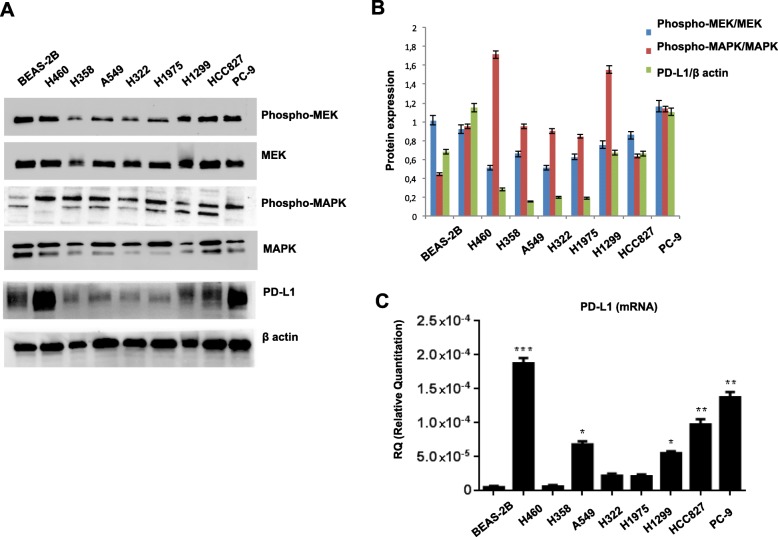
**a** Western blot analysis of MEK, phospho-MEK, MAPK, phospho-MAPK and PD-L1 on protein lysates from NSCLC cell lines HCC827, PC9, H1975, H460, H358, H322, H1299 and BEAS-2B. β-actin was included as a loading control. **b** Protein expression from densitometric analysis performed on three separate experiments. **c** Real time qPCR analysis of *PD-L1* mRNA expression. Results were normalized to 18S mRNA and analyzed by ΔCt method. One way ANOVA test followed by Tukey’s test were used for statistical analysis. * *p* < 0.05; **p < 0.01; ***p < 0.001

To assess the expression of PD-L1 in NSCLC, we performed analysis of both protein level, by western blot analysis (Fig. 1a-b), and of mRNA level, by RT-qPCR (Fig. 1c), in a panel of NSCLC cell lines, comparing them with BEAS-2B cell line, a human bronchial epithelial model. PD-L1 expression was heterogeneous across cell lines but the correlation between mRNA and protein level was consistent for any cell line, suggesting that ectopic PD-L1 expression mainly depends on transcriptional regulation. In the same models, we analyzed the activation status of the MAPK pathway (Fig. 1a, b) and we found that the majority of cells showed activated MAPK and MEK1/2 signals. Interestingly, the three cell lines in the panel with higher PD-L1 levels were HCC827 and PC9 cells, that are EGFR mutated, and H460, that is KRAS mutated, thus suggesting an interaction between intrinsic MAPK activation and PD-L1 expression.

**Fig. 2:**
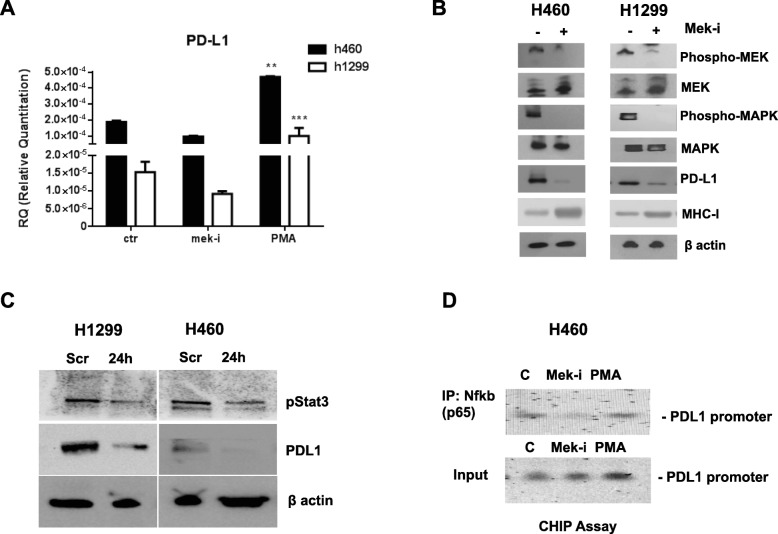
**a** Real time qPCR analysis of *PD-L1* mRNA expression in H460 and H1299 cell lines not treated (ctr), treated with selumetinib (mek-i) or stimulated with PMA (PMA). Results were normalized to 18S mRNA and analyzed by ΔCt method. One way ANOVA test followed by Tukey’s test were used for statistical analysis. **p < 0.01; ***p < 0.001. **b** Western blot analysis of MEK, phospho-MEK, MAPK, phopsho-MAPK, MHC-I and PD-L1 on protein lysates from NSCLC cell lines H460 and H1299 treated with selumetinib at indicated dose. β-actin was included as a loading control. **c** Levels of PD-L1 were measured in total protein extracts of H1299 and H460 cells that were transfected with scrambled (Scr) small interfering RNAs (siRNAs), or transfected with STAT3 siRNAs. β-Actin protein was used as a loading control for western blot analysis. **d** ChIP Assay evaluating the binding of NF-κB (p65) to the PD-L1 promoter in H1299 cells untreated or treated with MEK-i or PMA

Therefore, to better study the role of MAPK on PD-L1 expression regulation, we treated two selected cancer cell lines with the MEK-I, selumetinib: one with highest level of PD-L1, H460, and the other with intermediate levels of PD-L1, H1299. In those cells, we evaluated the changes in PD-L1 protein and mRNA expression after 24-h of treatment with 1 μM selumetinib and we recorded a significant decrease of PD-L1 levels (Fig. 2a, b, Additional file 2: Figure S2). To support the specificity of this result, we treated the cells with phorbol-12-myristate 13-acetate (PMA) stimulation, that directly activates MAPK, and we found a significant increase in PD-L1 mRNA levels (Fig. 2a). The change in mRNA levels of PD-L1 after MEK modulation suggests a transcriptional regulation on PD-L1 expression by MEK signal.

Since STAT3 is a MAPK downstream protein and has also a recognized role in mediating immune signals of interferon pathway, we performed a transient silencing of STAT3, to explore if it is involved in MAPK-dependent PD-L1 up-regulation. Results shown in Fig. 2c evidenced a proportional decrease of PD-L1 protein levels. To further analyze this effect, we studied the binding capability on the PD-L1 promoter of NF-kB (p65), a known transcription factor of MAPK and STAT3 signals, in the presence of selumetinib or PMA. ChIP analysis performed in H460 cells showed that the binding of Nf-kB(p65) is significantly decreased by selumetinib whereas increased by PMA (Fig. 2d).

### Role of MEK signal on tumor microenvironment signals

**Fig. 3:**
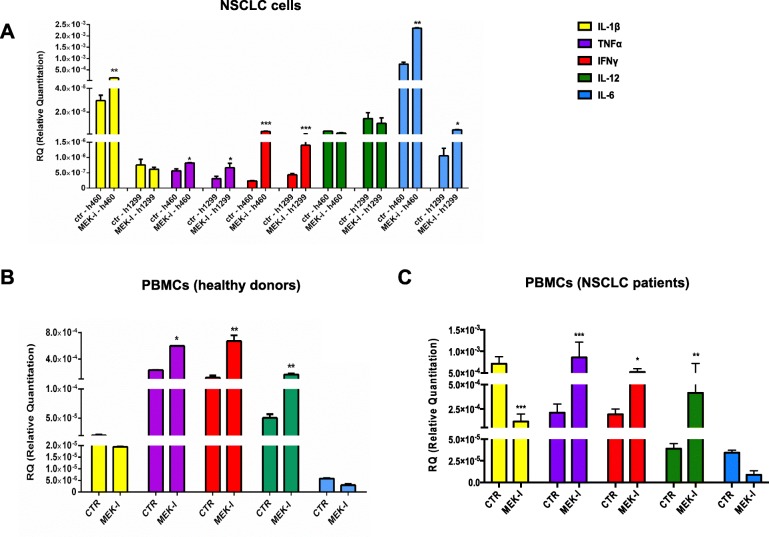
**a** Real time qPCR analysis of IL-1 β, TNFα, IFNγ AND il-6 expressed by H460 and h1299 cell lines not treated (CTR) or treated with selumetinib (MEK-i). Results were normalized to 18S mRNA and analyzed by ΔCt method. One way ANOVA test followed by Tukey’s test were used for statistical analysis. * *p* < 0.05; **p < 0.01; ***p < 0.001. **b**-**c**) IFNγ and IL-12 mRNA expression levels in PBMCs obtained from healthy donors (**b**) or NSCLC patients (**c**), stimulated for 5 days with anti CD3/CD28 beads. Real time qPCR analysis was conducted on cells cultured in absence (CTR) or presence of selumetinib (MEK-i). Results were normalized to 18S mRNA and analyzed by ΔCt method. One way ANOVA test followed by Tukey’s test were used for statistical analysis. * *p* < 0.05; **p < 0.01; ***p < 0.001

To analyze the effects of MEK inhibition on the immunogenicity of lung cancer cells, we analyzed the expression of both major histocompatibility complex class-I (MHC-I) by western blot and of several cytokines by RT-qPCR after treatment with selumetinib. MEK inhibition resulted in increased MHC-I expression on cancer cells (Fig. 2b), thus enhancing tumor recognition by immune system. In addition, MEK inhibition increased mRNA levels of IFN gamma, IL6, IL1B, and TNFα (Fig. 3a), all cytokines able to create a favorable microenvironment for inflammatory and immune response. Among cell lines, H1299 showed less upregulation of IL1B; since IL1B activation is dependent from caspase-1 protein that is regulated by p53, we speculate that p53 mutation impairs this process in H1299 cells, as compared to H460.

Similarly, we explored the effect of MEK-I on T-cell function, by using T-cells from PBMCs from healthy volunteers, activated with anti-CD3/anti-CD28 antibodies coated beads in terms of cytokine expression by RT-qPCR. MEK-I caused a significant increase of IL12 and IFNγ production after 5 days (Fig. 3b). Similar results were obtained using PBMCs from NSCLC patients (Fig. 3c).

### Efficacy of dual blockade of MEK and PD-L1 in ex vivo models

**Fig. 4:**
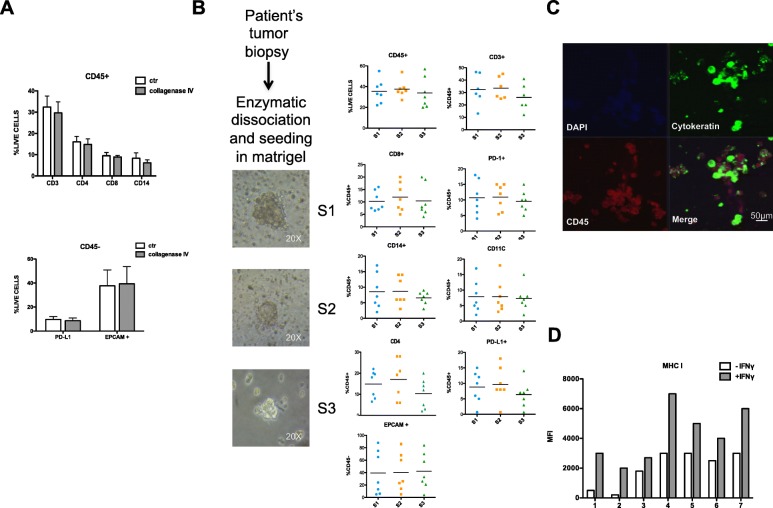
**a** Graphic representation of CD3, CD4, CD8 and CD14 expressed by PI negative and CD45 positive cells (up graph) and of PD-L1 and EPCAM expressed on CD45 and PI negative cells (down graph) obtained from lung biopsies digested with collagenase IV. The analysis was conducted by flow cytometry. **b** Schematic representation of biopsy enzymatic digestion; representative images of organoids obtained from each centrifugation (S1, S2, S3) are reported. Cells obtained from S1, S2 and S3 were analyzed by flow cytometric analysis. From PI negative and CD45 positive cells were analyzed markers like CD3, CD4, CD8, CD13, PD-1, CD11c and PD-L1. From cells negative for PI and CD45 were considered EPCAM+ cells. **c** Immunofluorescence analysis of spheroid stained with: DAPI, CD45 and Cytokeratin. **d** Graphic representation of MHC-I expressed on the surface of cancer cells before and after stimulation with IFNγ

We collected samples from lung tumors collected from NSCLC patients undergoing surgery or biopsy procedures at our Hospital and we processed them by enzymatic digestion in order to derive ex vivo 3D organoids primary cell cultures; they represent a valid model to study the effects of MEK-I on cancer cells and on the tumor microenvironment (Fig. 4), since they are multicellular organotypic spheroid cultures that preserve the inter-cellular interactions. In details, following the collagenase digestion process, they were seeded in matrigel and cultured with autologous immune cells.

Patients’ characteristics are reported in Additional file 3: Table S1**.** Majority of samples were obtained from surgical specimens (27% from stage I tumors, 27% from stage II, 9% from stage III) while 36% derived from biopsies from stage IV patients; in details, 18% taken from metastatic sites. Efficiency of establishment rate of primary culture in 3D substrate was lower in cultures derived from grade 1–2 tumors (33–60%) and from early stages (33–50%). PD-L1 expression was < 1% in 2/11 cases, 1–50% in 5/11 and > 50% in 4/11; we established ex-vivo cultures from 0% of PD-L1 negative cases, 60% of PD-L1 intermediate and 100% of high PD-L1 cases. 2/11 cases harbored *KRAS* mutations, and the 3D cultures from them were established.

We were able to establish 7/11 3D cultures with a total of 63.6% of successful establishment rate, which is similar to literature data [18–20]. Main difficulties in establishment of such models were represented by early death and low growth rate of tumor cells. However, in-vitro growth abilities of patient-derived 3D cultures were generally similar, by reaching a minimum diameter of 90 μm one week after seeding in matrigel (Fig. 4b) and continuing to grow for the following two weeks allowing drug testing.

After the enzymatic digestion, cells were analyzed by flow-cytometry to differentiate subpopulations included in the bulk tumor and then seeded in matrigel to generate spheroid cultures for exposure to treatments with anti-PD-L1 and/or MEK-I (Fig. 4). First, we compared the antigen expressions in bulk tumors versus digested fractions and we confirmed they were not altered by the enzymatic process (Fig. 4a). Then, we separated cells by filtration with three different filters (S1 > 100 μm; S2 30–100 μm; S3 < 30 μm) and we evaluated the lymphoid and myeloid immune cell fractions in each sample by flow-cytometry for specific antigens for any sub-populations (lymphoid: CD4^+^, CD8^+^; myeloid: CD14^+^, CD11c^+^; epithelial: EPCAM^+^) (Fig. 4b). Since S3 filtered spheroids were optimally sized, we utilized this fraction for subsequent studies. We further confirmed that tumor/immune cell mixture was preserved after digestion by immunofluorescence microscopy (Fig. 4c) for CD45 and EpCAM.

In addition, we analyzed MHC class I expression in cancer cells after stimulation with IFN**γ** and we found that all 7 ex vivo organoids cultures were formed by MHC class I proficient tumor cells (Fig. 4d).

**Fig. 5:**
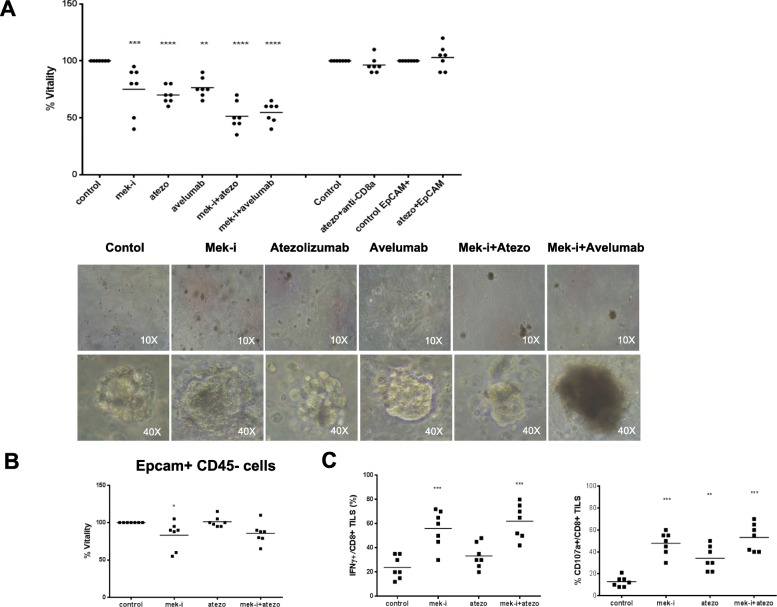
**a** MTT cell proliferation assays in human spheroids (A) or EPCAM+/CD45- cells (**b**), untreated or treated with selumetinib, atezolizumab, avelumab, or their combinations; (**c**) graphic flow cytometric analysis of IFNγ (left graph) or CD107A (right graph) produced by CD8+ TILs obtained from lung biopsies without treatments (CTR) or treated with selumetinib, atezolizumab or their combinations. One way ANOVA test followed by Tukey’s test were used for statistical analysis. * *p* < 0.05; **p < 0.01; ***p < 0.001

To evaluate response to blockade of PD-L1, MEK or both, we treated the 7 established ex vivo cultures with isotype control or an anti-PD-L1, atezolizumab or avelumab, or a MEK-I, selumetinib, or their combination for 3 and 6 days. Cell proliferation was quantified by MTT assay. Treatment with single agent atezolizumab or avelumab or selumetinib exerted a similar moderate anti-proliferative effect with ~ 30% cell death across all models, with the exception of the two KRAS mutated samples that resulted more sensitive to MEK-I (~ 50% cell death) (Fig. 5a). Although the magnitude of the response varied between patients, in all cases the combination of selumetinib and atezolizumab or avelumab obtained the strongest effect with a median of ~ 45% of cell death and ~ 60/55% in KRAS mutated cases (Additional file 1: Figure S1).

**Fig. 6:**
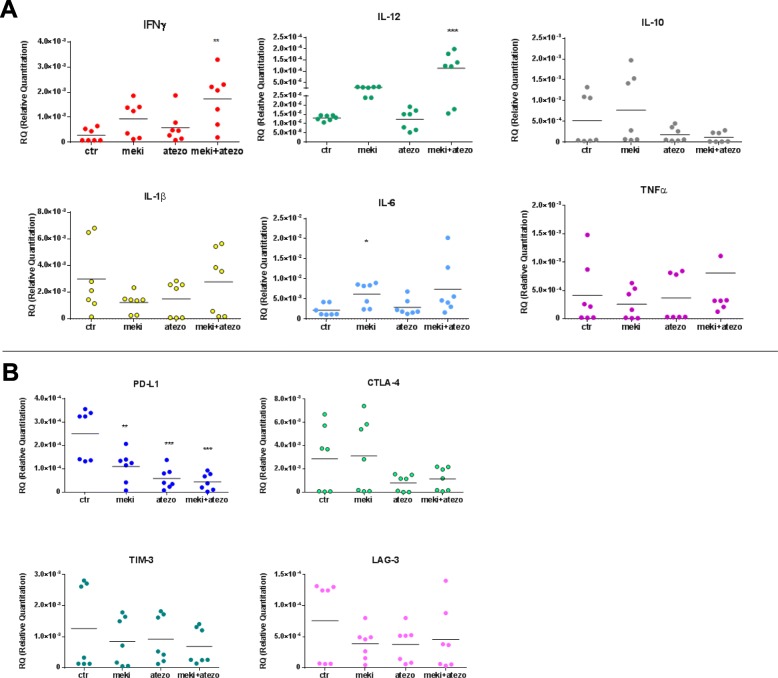
Real time qPCR analysis of cytochines IFNγ, IL-12, IL-10, IL-1 β, IL-6 and TNFα (**a**) or immune checkpoint genes as PD-L1, CTLA-4, TIM-3 and LAG-3 (**b**) expressed by spheroids untreated (CTR) or treated with selumetinib, atezolizumab or their combinations. Results were normalized to 18S mRNA and analyzed by ΔCt method. One way ANOVA test followed by Tukey’s test were used for statistical analysis. * *p* < 0.05; **p < 0.01; ***p < 0.001

The effect of anti-PD-L1 agent on cell viability requires immune cells and we demonstrated that the effect was likely due to immune cells killing of tumor cells by repeating the treatment with anti-PD-L1 atezolizumab in EPCAM^+^ subpopulation of spheroids cultures and confirming they were insensitive, since they lack the autologous immune cells (Fig. 5a). Moreover, atezolizumab effects were inhibited by co-treatment with an anti-CD8α antibody, demonstrating a specific requirement of CD8^+^ T cells (Fig. 5a).

On the other hand, we demonstrated that cell death induced by selumetinib was a consequence of both direct cell toxicity and increased immunoreactivity, as the lack of autologous immune/stromal cells or the addition of anti-CD8α antibody only partially influenced the effect of MEK-I (Fig. 5b).

To further confirm that the effect of treatment was due to increased tumor recognition by CD8^+^ T-cells, we demonstrated activation of CD8+ T-cells by quantifying the secretion of IFN**γ** and CD107a, a degranulation marker, after treatments (Fig. 5d).

Finally, we explored also the modification of cytokines and immune checkpoints gene expression by RT-PCR on spheroids after treatments and we detected an increase of IFNγ, IL12, IL6 and TNFα especially by combinations (Fig. 6a) and a simultaneous decrease of PD-L1, CTLA-4, TIM-3 and LAG-3 (Fig. 6b) thus indicating a potential role of MEK-I also on T cells exhaustion.

## Discussion

In the present work, we provide proof of concept that ex-vivo tumor organoids cultures can be used to establish individualized models to assess T cell-based therapies, thus representing a significant implementation for research in the field of immunotherapy of cancer. The use of this patient-specific derived model allows the study of interactions between tumor cells and T cells, recapitulating human leukocyte antigen (HLA) and T cell receptor (TCR) specific recognition. We foresee two major applications for this experimental platform. First, it is valid to explore both the anti-tumor efficacy of immunotherapy drugs and the effect of molecular targeted agents on both cancer- and T- cells. It implicates that this is a repeatable, simple and cheap model to test any immunotherapy drug in preclinical setting on the activation of T-cells. Since immunotherapy drugs are broadly used in cancer treatment worldwide and a lot of combination trials including immunotherapy drugs are ongoing, we speculate that the use of these models can replicate and predict in vivo clinical data. Second, it is useful to identify molecular pathways involved in sensitivity/resistance to immunotherapy, simply by protein and mRNA expression studies on different cell components of the organoids. Here, we identified by FACS analysisthat immune and cancer cells are co-present and are able to growth in this in-vitro system, thus confirming that digestion process is not affecting both viability and phenotypic characteristics of the different tumor components. This innovative aspect represents a straightforward research tool for translational studies on immunotherapy.

In the present study, using seven ex-vivo organoids cultures, we demonstrated a significant synergistic effect in terms of immune-dependent cancer cell death by the combination of MEK-I and anti-PD-L1 drugs. This synergism is the result of both direct cancer cell toxicity by MEK-I, as evidenced especially in two KRAS mutated samples that were the most sensitive ones to MEK-I, and the immune-stimulatory effect of MEK-I on cytokine secretion profile of cancer cells and PBMCs with the induction of all cytokines that are able to sustain an immune-reactive and inflammatory micro-environment. Mechanistically, this last aspect amplified even more the re-activation of T cells by anti-PD-L1 drugs. Previous studies have demonstrated a PD-L1 up-regulation in KRAS mutated cells [21], but the downstream pathways responsible for this are not fully elucidated. Chen et al., have demonstrated up regulation of PD-L1 in the context of KRAS mutation through ERK signal [22]. In a panel of NSCLC cell lines, we found that STAT3 is involved downstream in the transcriptional regulation of PD-L1 caused by RAS/MEK, thus providing another mechanistic rationale to combine MEK-I and anti-PD-L1 inhibitors. Moreover, we showed the upregulation of PD-L1 in-vitro by MEK-I and the synergism between MEK-I and anti-PD-L1 in different models, independently from KRAS mutation. This broadly shared mechanistic effect in unselected population suggests that MEK-I can have a role in all NSCLC patients as activator of immune response. However, considering that KRAS activating mutation is very frequent in NSCLC, accounting for about 30%, we think that additional studies are necessary to clarify if MEK-I can have also a more specific activity and to better identify which patients can benefit more from this combination therapy. In particular, among KRAS mutated NSCLC, there are various subgroups already known to be different in terms of proteomic and transcriptomic profiles, as established by Skoulidis et al. [23], including the KRAS/LKB1 mutated patients that represent an intrinsically resistant group to anti-PD-1/PD-L1 immunotherapies, with low immune and inflammatory marker expression, and the KRAS-only mutated and KRAS/TP53 mutated patients that are more sensitive to single agent immunotherapy treatments. We speculate that the addition of MEK-I to anti-PD-1/PD-L1 can be useful in KRAS mutated patients also to sensitize them to immunotherapy.

## Conclusions

Finally, there is a urgent need of novel combination strategies to prevent and overcome resistance to single-agent immunotherapies and to find biomarkers able to predict sensitivity to them. In this context, our result can be of high translational value since we identified the rationale for combining immunotherapy with MEK-I. Currently, combination of MEK-I and immunotherapy is in early clinical development in other cancer types, like breast and colon cancer (NCT03106415, NCT03374254). In addition, we showed a reproducible ex-vivo 3D culture model to study the effects of this combination. Additional studies should address deeply the molecular features of NSCLC that can be predictive of sensitivity to this combination strategy and also to explore novel personalized combinations between targeted agents and immunotherapy, especially for immune resistant subgroups of patients.

## Additional files

Additional file 1: **Figure S1.** MTT cell proliferation assays in two human spheroids harboring *KRAS* mutation untreated or treated with selumetinib, atezolizumab, avelumab, or their combinations. (PDF 80 kb)

Additional file 2: **Figure S2.** Protein expression from densitometric analysis performed on three separate experiments, for the western blot results showed in Fig. 2b. (PDF 76 kb)

Additional file 3: **Table S1.** Patients characteristics and establishment rate of 3D cultures (DOCX 50 kb)

## Data Availability

All data generated and analysed during this study are included in this published article and its Additional files 1, 2 and 3.

## Abbreviations

APC: Antigen-presentation cells
ATCC: American Type Culture Collection
BRAF-I: BRAF-inhibitor
ChIP: Chromatin immunoprecipitation
CTLA-4: Cytotoxic T-lymphocyte-associated antigen-4
HLA: Human leukocyte antigen
IP: Immunoprecipitation
ITT: Intention to treat
MEK-I: MEK inhibitor
MHC-I: Major histocompatibility complex class-I
MTT: 3-(4,5-dimethylthiazol-2-yl)-2,5-diphenyltetrazolium bromide
NSCLC: Non-small cell lung cancer
OS: Overall survival
PBMCs: Peripheral blood mononuclear cells
PBS: Phosphate-buffered saline
PD-1: Programmed cell death protein-1
PD-L1: Programmed cell death ligand-1
PFA: Paraformaldehyde
PMA: Phorbol-12-myristate 13-acetate
RT-qPCR: Real time quantitative PCR
SB: Staining buffer
siRNA: Small inhibitor duplex RNAs
TCR: T cell receptor
