# Supplementary figures and images for: Antitumor activity of dual blockade of PD-L1 and MEK in NSCLC patients derived three-dimensional spheroid cultures
Source: J Exp Clin Cancer Res. 2019 Jun 13;38:253. doi: 10.1186/s13046-019-1257-1 (PMC6567578; doi:10.1186/s13046-019-1257-1)

Supplemental figure 1: MTT assay on KRAS-mutated patients

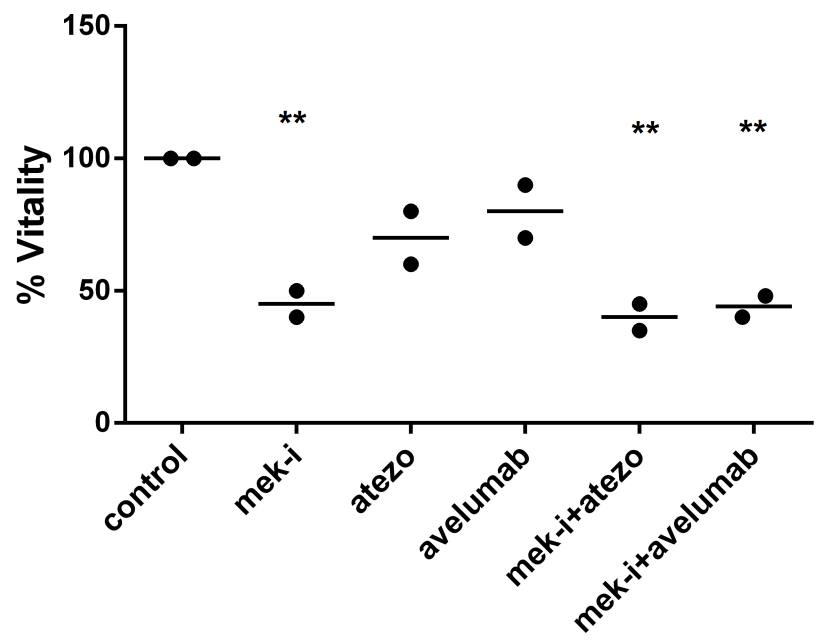

Supplement: Supplementary file 1 — Figure S1. MTT cell proliferation assays in two human spheroids harboring KRAS mutation untreated or treated with selumetinib, atezolizumab, avelumab, or their combinations. (PDF 80 kb) [file 13046_2019_1257_MOESM1_ESM.pdf]

Supplemental figure 2

A

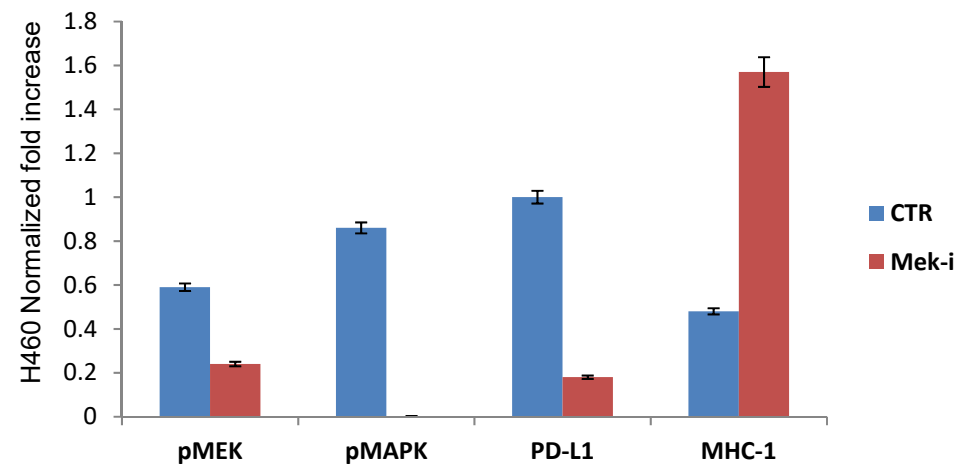

B

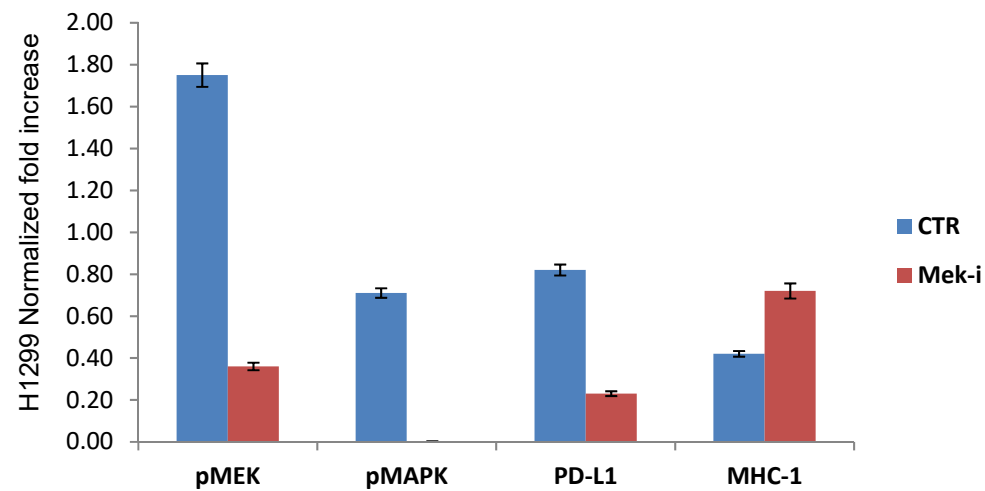

Supplement: Supplementary file 2 — Figure S2. Protein expression from densitometric analysis performed on three separate experiments, for the western blot results showed in Fig. 2b. (PDF 76 kb) [file 13046_2019_1257_MOESM2_ESM.pdf]
