# Supplementary material for: Antitumor activity of dual blockade of PD-L1 and MEK in NSCLC patients derived three-dimensional spheroid cultures
Source: J Exp Clin Cancer Res. 2019 Jun 13;38:253. doi: 10.1186/s13046-019-1257-1 (PMC6567578; doi:10.1186/s13046-019-1257-1)
Supplement: Supplementary file 3 — Table S1. Patients characteristics and establishment rate of 3D cultures (DOCX 50 kb) [file 13046_2019_1257_MOESM3_ESM.docx]

**Supplemental Table 1. Patients characteristics and establishment rate of 3D cultures**

| **Patient characteristics** | **Number** | **Establishment rate in 3D culture** |
| --- | --- | --- |
| **Stage** |  |  |
| **I** | **3** | **1** |
| **IIA** | **2** | **1** |
| **IIB** | **1** | **1** |
| **IIIA** | **1** | **1** |
| **IVA** | **3** | **2** |
| **IVB** | **1** | **1** |
| **Grading** |  |  |
| **1** | **3** | **1** |
| **2** | **5** | **3** |
| **3** | **3** | **3** |
| **Histology** |  |  |
| **Squamous** | **4** | **3** |
| **Nonsquamous** | **7** | **4** |
| **Sampling site** |  |  |
| **Primary** | **9** | **7** |
| **Metastatic site** | **2** | **1** |
| **PD-L1** |  |  |
| **<1%** | **2** | **0** |
| **1-50%** | **5** | **3** |
| **> 50%** | **4** | **4** |
| **Mutation/Rearrangment** |  |  |
| **EGFR** | **0** | **0** |
| **ALK** | **0** | **0** |
| **ROS** | **0** | **0** |
| **KRAS** | **2** | **2** |
